# Pediatric Alpha‐Fetoprotein‐Producing Gastric Cancer Presenting With Dysphagia and Multiple Liver Tumors

**DOI:** 10.1002/cnr2.70348

**Published:** 2025-09-09

**Authors:** Takayuki Hirano, Reina Hoshi, Bin Yamaoka, Kako Ono, Yosuke Watanabe, Shumpei Goto, Takashi Hosokawa, Koji Kanezawa, Katsuyoshi Shimozawa, Ayumu Arakawa, Shuichiro Uehara

**Affiliations:** ^1^ Department of Pediatric Surgery Nihon University School of Medicine Tokyo Japan; ^2^ Department of Pediatrics and Child Health Nihon University School of Medicine Tokyo Japan; ^3^ Department of Hematology and Oncology National Cancer Center Hospital Tokyo Japan

**Keywords:** alpha‐fetoprotein producing gastric cancer, children, gastrointestinal endoscopy, multiple liver tumors

## Abstract

**Background:**

Alpha‐fetoprotein (AFP)‐producing gastric cancer (AFPGC) is resistant to chemotherapy and is associated with poor prognosis. Pediatric gastric cancer has an incidence of 0.02% among gastric cancer patients, with a median survival of 5 months.

**Case:**

A 13‐year‐old boy presented with progressive dysphagia for 2 months, accompanied by loss of appetite and significant weight loss. Liver dysfunction and multiple lesions were observed. Laboratory investigations revealed elevated liver enzymes and tumor markers, including AFP, at 24 502 ng/mL. Computed tomography (CT) showed thickening of the gastric cardia and multiple liver lesions. Upper gastrointestinal endoscopy revealed a type 2 tumor in the cardia. Histopathology confirmed adenocarcinoma, and immunohistochemical staining was positive for AFP, establishing a diagnosis of AFPGC with liver metastases (Stage IV). Given the unresectable and HER2‐negative nature of the cancer, chemotherapy with TS‐1 and cisplatin was initiated, resulting in a temporary reduction in AFP levels and tumor size. However, disease progression was noted after 3 months, requiring a switch in treatment to ramucirumab, paclitaxel, bleomycin, etoposide, cisplatin, or a study drug. Despite these efforts, the patient succumbed to the disease 16 months after initial treatment.

**Conclusion:**

AFPGC in children is extremely rare, with few reported cases. The 16‐month survival observed in this case exceeds previously reported durations (8 and 4 months). Systematic evaluation of persistent gastrointestinal symptoms enabled earlier diagnosis. Standard adult gastric cancer treatment protocols appeared more effective than AFP‐tumor‐specific regimens, suggesting they may be optimal for pediatric AFPGC as well. Early diagnosis through detailed history‐taking and prompt endoscopic examinations in children with gastrointestinal symptoms may lead to significantly prolonged survival and improved management in this rare malignancy.

## 1 Introduction

Alpha‐fetoprotein (AFP)‐producing gastric cancer (AFPGC) is associated with chemotherapy resistance and poor prognosis [1]. AFPGC accounts for 2.7% to 5.4% of all gastric cancers [2, 3], and serum AFP levels vary across cases. One‐third of patients present with liver metastases at diagnosis, and overall prognosis is poor, with a 5‐year overall survival rate of approximately 20% [4]. Pediatric gastric cancer is exceedingly rare, with a frequency of 0.02% among gastric cancer patients [5]. Similarly, gastric cancer constitutes only 0.05% of all gastrointestinal malignant tumors in children [5]. The age of onset is typically over 10 years old [6], and the median survival time is 5 months, with a poor prognosis [7]. Symptoms are nonspecific, and upper gastrointestinal endoscopy—essential for diagnosis—is rarely performed in childhood, making early detection difficult. A nationwide survey conducted in Japan in 2016 identified only four pediatric cases of gastric cancer, three of whom died [8]. Additionally, Japan's vital statistics recorded only 15 gastric cancer‐related deaths in children aged < 15 years over a 30‐year period (1986–2015) [8].

Herein, we report a case of pediatric AFPGC presenting with dysphagia and multiple liver tumors. This case represents the third documented case of pediatric AFPGC and the first diagnosed prior to complications or rupture. The prolonged survival of 16 months, compared with previously reported cases, offers valuable insights into early diagnostic approaches and treatment strategies. Given the rarity and aggressive nature of pediatric AFPGC, understanding its clinical presentation, diagnostic challenges, and therapeutic responses in essential.

## 2 Case

This case was managed at Nihon University School of Medicine, Tokyo, Japan and National Cancer Center Hospital, Tokyo, Japan between April 2018 and August 2019. A 13‐year‐old boy presented with a chief complaint of progressive dysphagia lasting 2 months. He was previously healthy with no significant past medical history, family history of malignancy, or genetic predisposition to cancer. The patient's symptoms began insidiously with difficulty swallowing solid foods, which progressively worsened to include liquids. He developed appetite loss and experienced an 8% reduction in body weight, prompting a visit to a previous hospital. Liver dysfunction and multiple liver space‐occupying lesions were identified on abdominal ultrasonography, and the patient was referred to our department.

Physical examination revealed right hypochondrial bulging and tenderness, and the liver was palpable 7 cm below the costal margins. Laboratory findings showed mild anemia with a hemoglobin level of 12.1 g/dL. Elevated liver enzymes were noted, with aspartate aminotransferase/alanine aminotransferase levels of 98/91 IU/L and γ‐glutamyl transpeptidase levels of 488 U/L. Elevated levels of tumor markers were observed, with carcinoembryonic antigen levels of 8.4 ng/dL (normal < 5.0 ng/dL), protein induced by vitamin K absence or antagonist‐II levels of 28 413 mAU/mL (normal < 40 mAU/mL), and AFP levels of 24 502 ng/mL (normal < 10 ng/mL).

**FIGURE 1:**
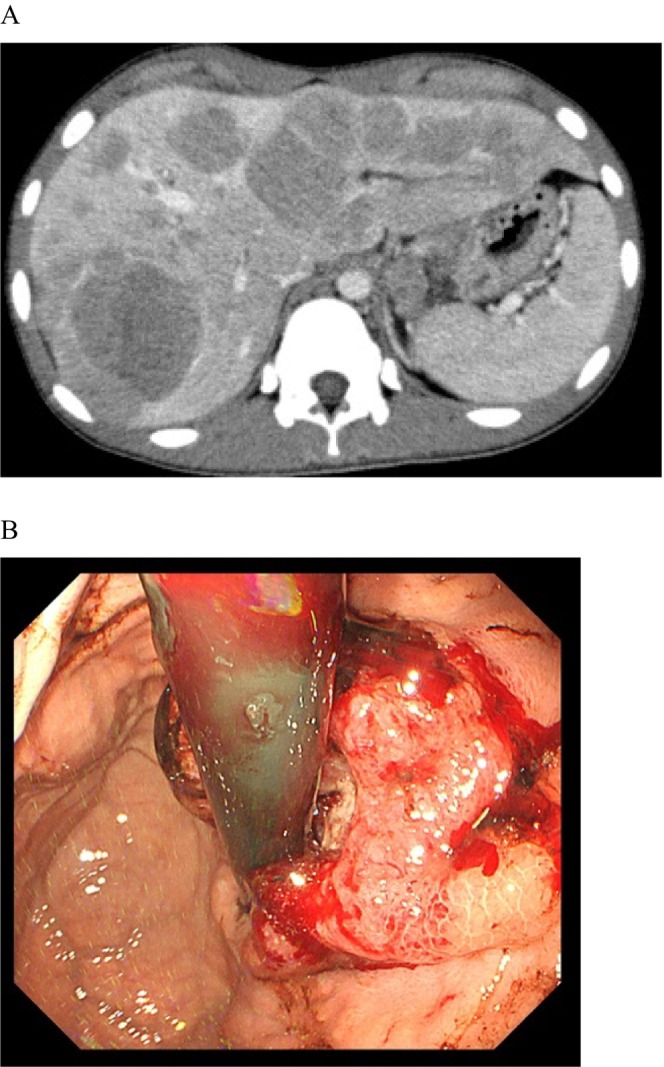
(A) CT tomography revealed mucosal thickening around the gastric cardia and enlarged lymph nodes on the lesser curvature. Multiple occupying lesions with ring enhancement were observed in both the lobes of the liver. (B) Gastrointestinal endoscopy revealed a 3/4 circumferential ulcerative lesion with a surrounding wall on the lesser curvature of the cardia. A tumor biopsy revealed moderately differentiated tubular adenocarcinoma and poorly differentiated adenocarcinoma.

**FIGURE 2:**
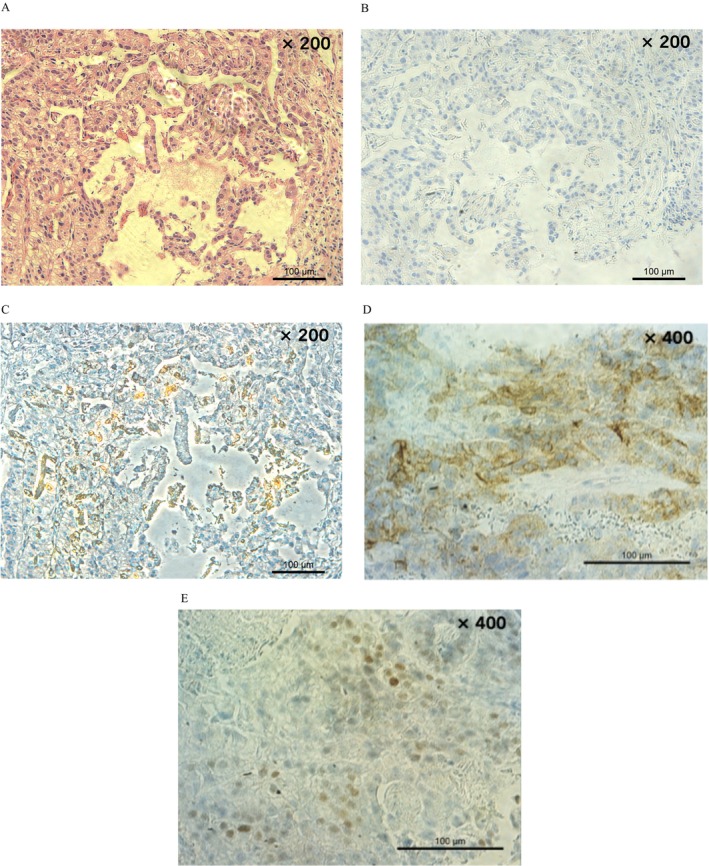
Microscopic appearance. (A) HE staining. Atypical cells with enlarged nuclei and increased chromatin form fused glandular structures, tubular structures with unclear lumen, and nested structures that proliferate invasively. (B) Immunohistochemistry (HER2). HER2 staining was negative. (C) Immunohistochemistry (AFP). AFP staining was positive in some of the cancer cells. (D) Immunohistochemistry (GPC 3). (E) Immunohistochemistry (SALL4). After second‐line treatment, a liver biopsy performed on liver metastases showed positive Glyptican3 and SALL4 staining.

Computed tomography (CT) showed thickening of the gastric cardia, enlargement of the regional lymph nodes, and multiple ring‐enhanced lesions throughout both liver lobes (Figure 1A). Gastrointestinal endoscopy performed to investigate the primary complaint of dysphagia revealed a type 2 tumor occupying 3/4 of the circumference in the gastric the cardia (Figure 1B). Tumor biopsy revealed poorly differentiated adenocarcinoma (Figure 2A). Additionally, immunohistochemical analysis revealed human epidermal growth factor receptor two (HER2) negativity and AFP positivity in numerous cancer cells (Figure 2B,C). Based on these findings, the patient was diagnosed with AFPGC and multiple liver metastases (cT3N1M1H1, Stage IV) [9].

## 3 Therapeutic Interventions

### 3.1 First‐Line Treatment

As the cancer was deemed unresectable with HER2‐negative status, chemotherapy with TS‐1 (tegafur/gimeracil/oteracil) and cisplatin was initiated according to adult gastric cancer guidelines [9]. This regimen achieved a temporary response: serum AFP levels decreased and the primary tumor shrank.

### 3.2 Second‐Line Treatment

However, 3 months after treatment initiation, the disease progressed. The chemotherapy regimen was changed to ramucirumab and paclitaxel combination. Although the disease was maintained for a while, it progressed again after 4 months.

### 3.3 Third‐Line Treatment

To explore alternative treatment options, a liver tumor biopsy of the metastatic lesion was performed 9 months after treatment initiation, revealing tumor cells with hyperchromatic atypical nuclei proliferating in papillary patterns and forming confluent glandular structures. Immunohistochemical staining was positive for AFP, Glypican3 (GPC3), and Sal‐like protein 4 (SALL4) (Figure 2D,E). Based on these results and considering the AFP‐producing nature and potential germ cell tumor differentiation, bleomycin, etoposide, and cisplatin (BEP) therapy was initiated 12 months after initial treatment, following the protocol for germ cell tumors (GCTs).

### 3.4 Fourth‐Line Treatment

Fourteen months after the initial treatment, the study drug was initiated; however, there was no response.

## 4 Follow‐Up and Outcomes

The patient was monitored with regular clinical assessments, laboratory studies (including AFP levels), and imaging studies. Despite multimodal treatment approaches, the patient experienced progressive disease with increasing tumor burden and AFP levels. Supportive care was intensified during the final months, and the patient died 16 months after treatment initiation due to progressive disease and liver failure.

## 5 Discussion

This case represents a significant contribution to pediatric oncology literature as the third reported instance of pediatric AFPGC and the first diagnosed prior to catastrophic complications. The 16‐month survival observed in this case is the longest reported to date, exceeding previously documented survival durations of 8 and 4 months [10, 11].

Two cases of pediatric AFPGC have been reported, both involving boys. The first case involved a 12‐year‐old who presented with gastric perforation [10]. After undergoing surgery to repair the perforation, he was readmitted with peritoneal dissemination. CT imaging revealed gastric wall thickening, and the diagnosis was established through exploratory laparotomy and upper gastrointestinal endoscopy. He died 8 months after the initial presentation. The second case involved a 14‐year‐old boy who presented with a ruptured metastatic liver tumor [11]. A liver biopsy failed to provide a diagnosis, and a right liver lobectomy was performed for definitive diagnosis. Histological analysis suggested a tubular adenocarcinoma of gastrointestinal origin, and the final diagnosis was confirmed through upper gastrointestinal endoscopy. He died 4 months after initial presentation. In contrast, the 13‐year‐old boy in our case was diagnosed through systematic evaluation of dysphagia, without complications such as perforation or rupture, and achieved a survival duration of 16 months.

In the histological classification of AFPGC, subtypes such as hepatoid adenocarcinoma, fetal gastrointestinal epithelium‐like carcinoma, and yolk sac tumor‐like carcinoma have been reported [1]. In addition to AFP, overexpression of other markers—including GPC3 and SALL4—has also been observed in these tumors [12]. GPC3 is a cell surface protein belonging to the heparan sulfate proteoglycan family and is believed to play a role in regulating cell proliferation, differentiation, and migration [13]. It is notably expressed in hepatoid carcinomas, where it contributes to the regulation of cell growth [14]. SALL4 is a transcription factor essential for fetal organ development and is involved in maintaining and regulating stem cell differentiation. It is highly expressed in embryonic stem cells, hematopoietic stem cells, and certain cancer cells. High SALL4 expression has been reported in hepatoid and enteroblastic adenocarcinomas [15]. Moreover, SALL4 has been identified as a marker for a progenitor subclass of HCC with an aggressivephenotype [16, 17]. Although AFP is primarily produced by the fetal liver and yolk sac, its expression has been confirmed in the fetal gastrointestinal tract [18]. This suggests that gastrointestinal cancers may produce a distinct form of AFP.

The immunohistochemical profile of our case, demonstrating positivity for AFP, GPC3, and SALL4, suggests enteroblastic differentiation. In addition to the tubular and papillary proliferation patterns observed in the gastric tumor biopsy obtained via upper gastrointestinal endoscopy, immunohistochemical staining of a liver tumor biopsy—performed 9 months after the initiation of treatment—also showed positivity for AFP, GPC3, and SALL4. These fetal protein markers indicate potential dedifferentiation or primitive differentiation of gastric tissue toward an enteroblastic phenotype. Their expression in AFPGC is known to overlap with that of fetal gastrointestinal epithelium‐like cancer [12, 19]. This supports existing theories suggesting that the pathogenesis of AFPGC may involve embryological reversion.

No established chemotherapy regimen exists for AFPGC [9], and it is common practice to follow adult gastric cancer protocols. In our case, treatment included standard gastric cancer protocols (which proved most effective), GCT protocols (considering AFP production), and investigational agents. Differentiation from GCTs is particularly important in pediatric AFPGC. Considering the possibility of malignant transformation from a GCT, BEP therapy was administered; however, it proved ineffective. The temporary response to TS‐1 and cisplatin suggests that adult gastric cancer regimens may represent the optimal first‐line approach for pediatric AFPGC, rather than protocols designed for other AFP‐producing tumors.

Several factors may have contributed to the extended 16‐month survival compared with previously reported pediatric cases. First, systematic evaluation of dysphagia led to diagnosis before the onset of catastrophic complications such as perforation or rupture. In general, pediatric liver tumors are initially assessed via biopsy [20, 21]. but in older children outside the typical age range for hepatoblastoma, diagnosis may be challenging and appropriate chemotherapy may be delayed. Second, the sequential use of different treatment modalities based on tumor response and biopsy findings proved beneficial. Third, coordinated multidisciplinary care—including pediatric oncology, gastroenterology, and surgical teams—enhanced the overall management of the patient.

Several clinical considerations emerge from this case. Persistent gastrointestinal symptoms in adolescents warrant thorough evaluation, including endoscopy when indicated. Prioritizing the patient's chief complaints can support earlier diagnosis, even in rare conditions. In terms of treatment, adult gastric cancer protocols may offer greater efficacy than AFP‐tumor‐specific regimens in pediatric AFPGC.

This case report documents the longest‐surviving pediatric patient with AFPGC reported to date. The key clinical message is that systematic evaluation of persistent gastrointestinal symptoms—particularly dysphagia—can facilitate early diagnosis of rare malignancies before life‐threatening complications arise. For pediatricians, this case underscores the importance of maintaining clinical suspicion for rare cancers when adolescents present with persistent, unexplained gastrointestinal symptoms. Early endoscopic evaluation and prompt initiation of appropriate chemotherapy protocols may improve patient outcomes. Given the extreme rarity of pediatric AFPGC, international collaboration and centralized case registries may be essential to advancing understanding of optimal treatment strategies and identifying prognostic factors to guide therapeutic decision‐making.

The overall significance of this report lies in demonstrating that early diagnosis and timely treatment of pediatric AFPGC, combined with flexible adjustments to chemotherapy regimens, can meaningfully prolong survival—even if cure is not achievable—compared with cases diagnosed only after severe complications have developed.

## Author Contributions

**Reina Hoshi:** writing, reviewing, and editing. **Takayuki Hirano:** validation, conceptualization, writing, original draft, writing, review, and editing. **Bin Yamaoka:** writing, reviewing, and editing. **Kako Ono:** writing, review, and editing. **Yosuke Watanabe:** writing, reviewing, and editing. **Shumpei Goto:** writing, reviewing, and editing. **Takashi Hosokawa:** writing, reviewing, and editing. **Koji Kanezawa:** writing, reviewing, and editing. **Katsuyoshi Shimozawa:** writing, reviewing, and editing. **Ayumu Arakawa:** validation, investigation, resources, writing, review, and editing. **Shuichiro Uehara:** writing, reviewing, editing, and supervision.

## Ethics Statement

The patient's next of kin provided informed consent for publication of the case report.

## Consent

The patient's next of kin provided informed consent for publication of the case report.

## Conflicts of Interest

The authors declare no conflicts of interest.

## Data Availability

Data sharing not applicable to this article as no datasets were generated or analysed during the current study.
